# The phosphorylated regulator of chemotaxis is crucial throughout biofilm biogenesis in *Shewanella oneidensis*

**DOI:** 10.1038/s41522-020-00165-5

**Published:** 2020-11-13

**Authors:** Anne Boyeldieu, Amine Ali Chaouche, Moly Ba, Flora Ambre Honoré, Vincent Méjean, Cécile Jourlin-Castelli

**Affiliations:** 1 Laboratoire de Bioénergétique et Ingénierie des Protéines (BIP, UMR7281), Aix Marseille Université, Centre National de la Recherche Scientifique, Marseille, France; 2 Laboratoire d’Ingénierie des Systèmes Macromoléculaires (LISM, UMR7255), Aix Marseille Université, Centre National de la Recherche Scientifique, Marseille, France; 3 Present Address: Laboratoire Information Génomique et Structurale (IGS, UMR7256), Aix Marseille Université, Centre National de la Recherche Scientifique, Marseille, France

**Keywords:** Biofilms, Microbial genetics

## Abstract

The core of the chemotaxis system of *Shewanella oneidensis* is made of the CheA3 kinase and the CheY3 regulator. When appropriated, CheA3 phosphorylates CheY3, which, in turn, binds to the rotor of the flagellum to modify the swimming direction. In this study, we showed that phosphorylated CheY3 (CheY3-P) also plays an essential role during biogenesis of the solid-surface-associated biofilm (SSA-biofilm). Indeed, in a ΔcheY3 strain, the formation of this biofilm is abolished. Using the phospho-mimetic CheY3D56E mutant, we showed that CheY-P is required throughout the biogenesis of the biofilm but CheY3 phosphorylation is independent of CheA3 during this process. We have recently found that CheY3 interacts with two diguanylate cyclases (DGCs) and with MxdA, the c-di-GMP effector, probably triggering exopolysaccharide synthesis by the Mxd machinery. Here, we discovered two additional DGCs involved in SSA-biofilm development and showed that one of them interacts with CheY3. We therefore propose that CheY3-P acts together with DGCs to control SSA-biofilm formation. Interestingly, two orthologous CheY regulators complement the biofilm defect of a ΔcheY3 strain, supporting the idea that biofilm formation could involve CheY regulators in other bacteria.

## Introduction

Bacteria can adopt either a planktonic or sessile lifestyle. Both are highly regulated, as is the switch from one to another. During the planktonic lifestyle, the flagellated bacteria move around in their environment. They adapt the orientation of their movement depending on their surroundings, going toward favorable niches (attractants) and moving away from unfavorable or toxic ones (repellents). This mechanism of oriented swimming is called chemotaxis and is governed by a complex but well studied regulatory machinery, named the Che system^1–3^. The latter is a sophisticated two-component regulatory system centered around the histidine kinase-response regulator couple, consisting of CheA and CheY. The autophosphorylation of CheA depends on signal detection via the methyl-accepting chemotaxis proteins (MCP) that form highly organized complex arrays located in the membrane and which are connected to CheA via the CheW proteins^4–6^. CheA-P then serves as a phosphodonor for the CheY regulator. Once phosphorylated, CheY interacts with the motor of the flagellum to modulate its rotation, enabling the chemotactic behavior.

During the sessile lifestyle, bacteria live inside community structures called biofilms^7^. In these structures, the cells are embedded in a self-produced matrix that commonly contains exopolysaccharides, proteins, and DNA^8^. Previously, the term biofilm only referred to the biofilm formed by the cells on surfaces. The latter is now called the solid-surface-associated biofilm (SSA-biofilm) to distinguish it from other types of biofilms. The floating biofilm, commonly called the pellicle, is a biofilm that the cells form at the air–liquid interface, where they benefit from both the nutrient underneath and the above oxygen^9,10^. Another type of biofilm that is being increasingly studied is the macrocolony biofilm that is formed on nutrient agar^11^. The development of these biofilms is controlled at the transcriptional to post-translational levels by several regulatory mechanisms. Among the well-known mechanisms often encountered in biofilm control are the two-component regulatory systems and the quorum-sensing mechanisms, as well as small RNA, riboswitches, and alternative sigma factors. For example, in *Pseudomonas aeruginosa*, the transcriptional factor FleQ is a master regulator controlling the expression of flagellar and exopolysaccharide biosynthesis genes^12,13^. Another example is the complex regulatory network involving several histidine kinases which control the expression of two small non-coding RNAs (RsmY and RsmZ). Once expressed, these sequester the translational repressor RsmA, enabling biofilm formation^14–16^.

The switch from the planktonic to the sessile lifestyle is regulated by a bacterial second messenger, the cyclic diguanylate monophosphate (c-di-GMP)^17,18^. The concentration of c-di-GMP in cells depends on two types of enzymes with antagonistic actions, the diguanylate cyclases (DGCs) that synthesize it and the phosphodiesterases (PDEs) that hydrolyze it. The DGCs contain a characteristic “GGDEF” domain containing a consensual GG(D/E)EF active site, while the PDEs harbor either an EAL or a HD-GYP domain^19^. To trigger its effects in the cells, the synthesized c-di-GMP can bind to either mRNA (riboswitches) or effector proteins. The latter is far more difficult to identify than DGCs and PDEs, since several domains have been shown to serve as binding sites for c-di-GMP with others still to be found^20^. Bacterial genomes often contain a high number of DGC and PDE encoding genes, as well as several genes coding for effector proteins. This observation raised major questions, including why so many and how the specificity of the response can be ensured. In fact, certain DGC and PDE genes are expressed in response to specific signals and the corresponding proteins are usually active only under specific conditions. However, another layer of control was more recently discovered which involves c-di-GMP local signaling. The basis of this mechanism is found in the partnership between a DGC and an effector protein, ensuring that the c-di-GMP synthesized is directly transferred to the target effector, and therefore triggering an appropriate response. For example, in *Pseudomonas fluorescens*, the diguanylate cyclase GcbC interacts with an effector protein called LapD, which promotes biofilm formation upon c-di-GMP binding^21^. Very recently, this local signaling was also discovered in *Bacillus subtilis*^22^. Specific interactions leading to the delivery of c-di-GMP to the target effectors is discussed in detail in Dahlstrom and O’Toole^23^.

*Shewanella oneidensis* is an aquatic bacterium which is well known for its wide range of respiratory systems, enabling it to thrive in various environments^24,25^. However, its adaptive capabilities are not restricted to its respiratory versatility. Indeed, *S. oneidensis* is motile, using a unique polar flagellum and is capable of chemotactic behavior in response to several molecules^26–29^. Its genome contains three loci harboring *che* genes. The *che1* locus encodes a chemosensory Che-like system involved in the post-translational regulation of RpoS by a partner switch mechanism^30^. The *che2* locus is probably not functional, due to IS insertions into *cheA2* and an associated MCP-encoding gene. The third locus (*che3*) is immediately downstream of the genes encoding the flagellum apparatus and the σ^28^ factor controlling late gene expression. Accordingly, the Che3 system was shown to control chemotaxis in *S. oneidensis*, as either a *cheA3* or a *cheY3* mutant is non-chemotactic^28,31,32^. Finally, *S. oneidensis* is able to form two types of biofilm. *S. oneidensis* was first shown to form an SSA-biofilm following a common set of steps from initial surface attachment to microcolony formation and then to three-dimensional structures^33^. Initial attachment requires the mannose-sensitive hemagglutinin type IV pilus, while swimming motility contributes to the 3D-structure development^33,34^. Interestingly, it was shown that the majority of cells are metabolically active inside the biofilm^35^. Although the composition of the biofilm matrix is not totally characterized, it was shown that extracellular DNA is a crucial factor for SSA-biofilm development^36^. Moreover, the *mxdABCD* gene cluster was proven to be required for the formation of the 3D-structure, as *mxd* mutants formed a flat SSA-biofilm^34,37^. Given that MxdB harbors a glycosyl transferase domain, it was proposed that the Mxd machinery could be involved in the biosynthesis of an exopolysaccharide. Finally, the involvement of c-di-GMP in SSA-biofilm regulation was demonstrated^37^. However, while a phosphodiesterase (PdeB) was shown to be implicated in the control of the c-di-GMP pool, no specific DGC has yet been shown to be involved in SSA-biofilm biogenesis in *S. oneidensis*^38^.

More recently, *S. oneidensis* was shown to form a cohesive and homogeneous pellicle at the air–liquid interface, when grown under static conditions^32,39^. Pellicle formation only happens in the presence of oxygen. Localization to the air–liquid interface is an active process requiring flagellated and hence motile cells^32^. Moreover, we showed that the chemotaxis Che3 system is involved in the control of pellicle biogenesis^32^. Indeed, a *cheY3*-deleted strain is not able to form a pellicle, while the *cheA3*-deleted strain forms a heterogeneous and non-cohesive pellicle. Recently, we identified two DGCs implicated in pellicle formation, PdgA, and PdgB, and showed that they interact with CheY3. Interestingly, PdgA also interacts with MxdA that proved to be a c-di-GMP-binding protein^40^. This partnership DGC-effector protein strengthens the c-di-GMP local signaling model.

In this work, we showed that all steps of the SSA-biofilm biogenesis require the phosphorylated form of CheY3. We also identify two novel SSA-biofilm DGCs and one which interacts with CheY3. As orthologous CheY can replace CheY3 for SSA-biofilm biogenesis, we propose that the chemotaxis regulator CheY could also play a key role during SSA-biofilm development in other bacteria.

## Results

### CheY3 is mandatory for SSA-biofilm formation

**Fig. 1:**
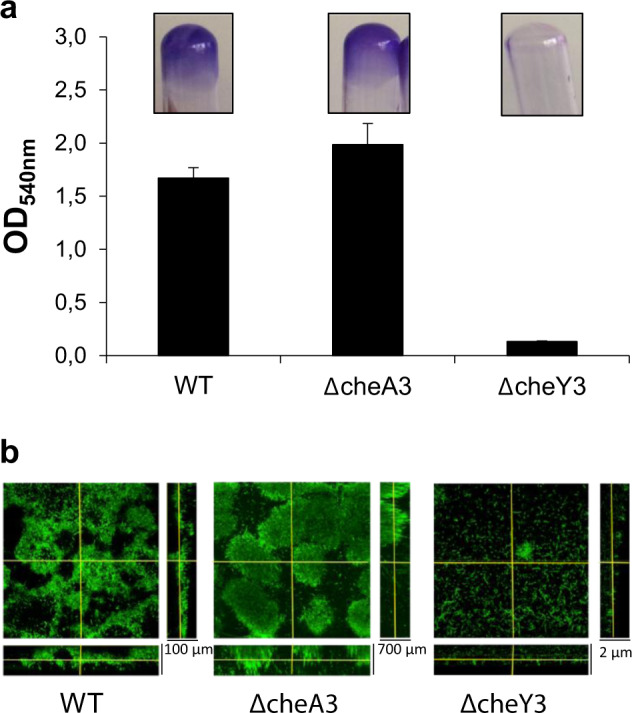
SSA-biofilm formation requires the CheY3 chemotaxis response regulator. **a** Biofilm formation in glass tubes. The wild-type strain (WT), the ΔcheA3 and ΔcheY3 mutant strains were grown at 28 °C in LM medium containing 20 mM lactate in glass tubes under shaking conditions. After 24 h of incubation, biofilm formation was evaluated by crystal violet staining, photographed, and quantified by OD_540nm_ measurements. The means and standard deviations from two independent experiments conducted in duplicate are shown. **b** Biofilm formation in flow cells. The wild-type and mutant strains containing the pX5-GFP plasmid were grown in three-channel flow cells in LM medium containing 20 mM lactate for 24 h. Biofilms were analyzed by confocal laser scanning microscopy and corresponding extracted z images and their respective xy and xz planes are presented.

Given that the CheA3 kinase and the CheY3 chemotaxis regulator of *S. oneidensis* play a key role during pellicle formation (see above), we looked for their possible role in SSA-biofilm biogenesis. Therefore, the wild-type, ΔcheA3, and ΔcheY3 strains were grown in LM medium containing lactate under shaking conditions for 24 h. The cells that adhered to the tube walls were then stained with crystal violet (CV). As shown in Fig. 1a, both the wild-type and the ΔcheA3 strains were able to form a biofilm. CV-quantification seemed to indicate that the biomass of the ΔcheA3 biofilm is slightly higher than that of the wild-type one. On the contrary, the ΔcheY3 strain was entirely unable to form an SSA-biofilm (Fig. 1a).

To ascertain these striking results, we performed a similar experiment under hydrodynamic conditions using flow cell chambers. As previously shown by others, the biofilm formed by the wild-type strain covered most of the glass surface and showed a usual 3D-structure (Fig. 1b). In accordance with the above assumption, the ΔcheA3 strain also formed a biofilm and featured taller mushroom structures compared to that of the wild-type. Finally, the ΔcheY3 strain showed no biofilm structure, and only a few cells were visible under the microscope, most of which were motile (Fig. 1b).

Interestingly, these results clearly indicate that the CheY3 chemotaxis regulator is absolutely required for SSA-biofilm biogenesis. In contrast to pellicle formation, the CheA3 kinase does not play a major role in SSA-biofilm formation.

### CheY3 must be phosphorylated to allow SSA-biofilm formation

**Fig. 2:**
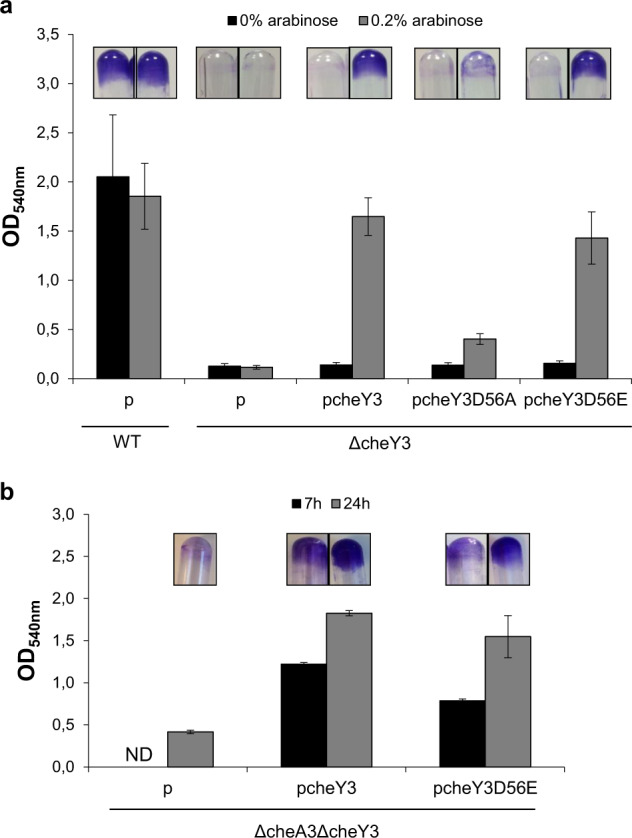
Biofilm formation involves the phosphorylation of CheY3 independent of the CheA3 kinase. **a** Glass-tube biofilm assays of the wild-type and the ΔcheY3 strains containing either the empty vector (p) or the pcheY3 WT or mutant plasmids. Biofilms were grown for 24 h with (gray bars) or without (black bars) 0.2% arabinose for 24 h before CV-staining, imaging, and quantification. **b** Glass-tube biofilm assays of the ΔcheA3ΔcheY3 mutant strain containing either the empty vector (p) or the pcheY3 WT or mutant plasmids. Biofilms were grown for 7 h (black bars) or 24 h (gray bars) with 0.2% arabinose before CV-staining, imaging, and quantification. ND: not determined. The means and standard deviations from two independent experiments conducted in duplicate are shown.

We next wanted to determine whether the phosphorylated or non-phosphorylated form of CheY3 was required for SSA-biofilm formation. For this purpose, we used two mutated alleles of *cheY3* encoding a phospho-ablative and a phospho-mimetic CheY3, in which the phosphorylatable aspartate residue (position 56) was replaced by either an alanine or a glutamate, respectively. The two mutated alleles, along with the wild-type one, were placed under the control of an arabinose-inducible promoter and introduced into the ΔcheY3 strain. As expected, the ΔcheY3 strain containing the wild-type *cheY3* allele in trans was able to form an SSA-biofilm comparable to the wild-type strain, in the presence of arabinose (Fig. 2a). Unlike its wild-type counterpart, the *cheY3D56A* allele encoding a non-phosphorylatable form of CheY3 was unable to restore SSA-biofilm formation in the ΔcheY3 background. Strikingly, the *cheY3D56E* allele was able to complement the ΔcheY3 strain and allowed the formation of an SSA-biofilm similar to that of the ΔcheY3 strain complemented with the wild-type *cheY3* allele (Fig. 2a).

These results strongly suggest that the CheY3 active form allowing SSA-biofilm formation is the phosphorylated one. In contrast, for pellicle formation, neither the *cheY3D56A* nor the *cheY3D56E* allele was able to complement the ΔcheY3 strain^32,40^.

It is well known that CheA3 is the kinase partner of CheY3 for chemotaxis. As CheA3 does not seem to be required for SSA-biofilm formation, and thus probably does not phosphorylate CheY3 during this process, we tested the behavior of the double ΔcheA3ΔcheY3 mutant strain complemented or not with *cheY3* alleles. As shown in Fig. 2b, the ΔcheA3ΔcheY3 strain is unable to form a biofilm, as reported for the ΔcheY3 strain. However, the presence of either the wild-type *cheY3* or *cheY3D56E* allele allowed the restoration of biofilm formation.

These results confirm that CheY3 phosphorylation is not carried out by CheA3 during the biogenesis of the SSA-biofilm. Together, our results reveal that phosphorylated CheY3 is essential for SSA-biofilm formation and that, consequently, phosphodonors such as histidine kinases other than CheA3, are required for this process.

### The phosphorylation of CheY3 is required throughout SSA-biofilm formation

**Fig. 3:**
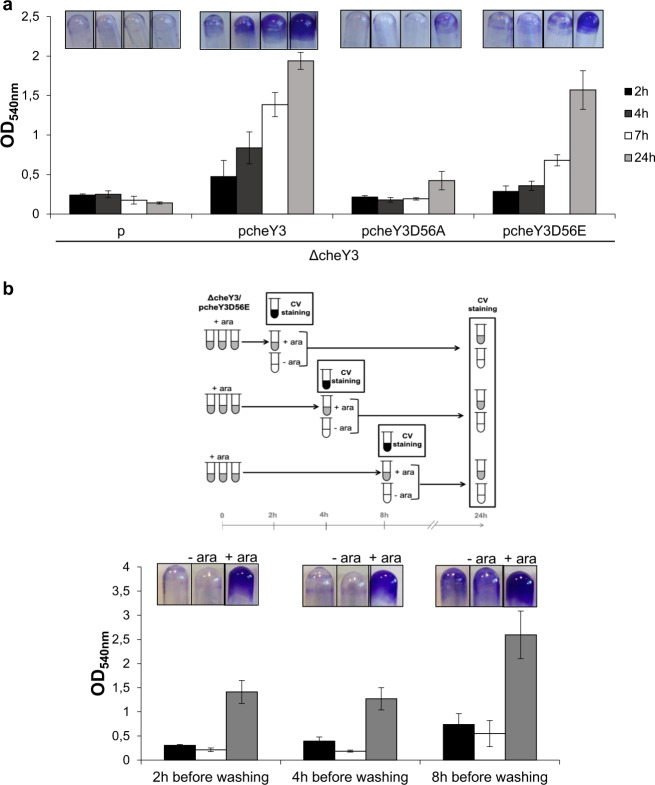
Phosphorylated CheY3 is required throughout biofilm formation. **a** Glass-tube biofilm assays of the ΔcheY3 strain containing either the empty vector (p) or the pcheY3 WT or mutant plasmids. The strains were grown for 2 h (black bars), 4 h (dark gray bars), 7 h (white bars), and 24 h (light gray bars) with 0.2% arabinose before CV-staining, imaging, and quantification. The means and standard deviations from two independent experiments conducted in duplicate are shown. **b** Kinetics of biofilm formation in glass tubes. On the top, the experimental strategy is schematized. At *t* = 0 h, the ΔcheY3 strain containing pcheY3D56E plasmid was incubated in SSA-biofilm formation conditions with 0.2% arabinose. After 2, 4, and 8 h of pre-incubation, one test tube was CV-stained, imaged, and quantified (black tubes and black bars). Two other tubes were emptied, washed with LM medium, and refilled with the ΔcheY3/pcheY3D56E strain in LM medium containing 20 mM lactate in the absence (white tubes) or presence of arabinose (gray tubes). These tubes were incubated in SSA-biofilm forming conditions. At *t* = 24 h, all samples containing 0% (white tubes and white bars) and 0.2% arabinose (gray tubes and gray bars) were CV-stained, imaged, and quantified. The means and standard deviations from two independent experiments conducted in duplicate are shown.

We wondered whether CheY3 must be phosphorylated from the early to the late steps of biofilm formation or whether phosphorylation is only required for some steps. To answer this question, we first followed biofilm formation in the ΔcheY3 strain complemented or not with a *cheY3* allele. As shown in Fig. 3a, in the presence of the wild-type *cheY3* allele, a significant amount of biofilm was formed after 2 h of incubation, which increased over time. Unlike its wild-type counterpart, the *cheY3D56A* allele did not allow the initiation of biofilm formation, as the biofilm quantification from 2 to 7 h was identical to that obtained in the absence of a *cheY3* allele (empty vector). Moreover, only a very small amount of biofilm was produced after 24 h of incubation in the presence of this mutated allele (Figs. 2a and 3a). In contrast, the presence of the *cheY3D56E* allele allowed the initiation of biofilm formation. Indeed, the biofilm amount was higher overall in the presence of the *cheY3D56E* allele than in the control with no *cheY3* allele, and increased over time. This is reminiscent of that which occurs in the presence of the wild-type *cheY3* allele, even if the increase is slightly lower in the presence of *cheY3D56E* (Fig. 3a). These results indicate that the phosphorylation of CheY3 is required for biofilm initiation to occur.

To determine whether CheY3 phosphorylation is still required once biofilm formation has initiated, we performed further experiments by controlling the time-frame of *cheY3D56E* expression. The ΔcheY3 strain complemented with the *cheY3D56E* allele was grown in triplicate tubes for 2, 4, or 8 h in the presence of arabinose, allowing expression of the *cheY3D56E* allele. One tube was then CV-stained and used to quantify the extent of biofilm formation at the corresponding time. The other two tubes were emptied, washed, and re-inoculated with the same strain in the presence or absence of arabinose, respectively, allowing or not the expression of the *cheY3D56E* allele, and were incubated for a maximum of 24 h. As shown in Fig. 3b, when arabinose was omitted from the medium after the washing step, biofilm formation stopped and the amount of biofilm was similar to or lower than that obtained before the washing step. On the contrary, when arabinose was added after the washing step, biofilm formation continued to increase. These results indicate that CheY3 phosphorylation is required after the initiation steps have taken place.

Altogether, this strongly suggests that CheY3 must be phosphorylated from the early to the late steps of SSA-biofilm biogenesis and that CheY3-P is the active form of CheY3, which plays a key role during the steps of biofilm development.

### Overexpression of two diguanylate cyclase-encoding genes restores SSA-biofilm formation in the *cheY3*-deleted mutant strain

**Fig. 4:**
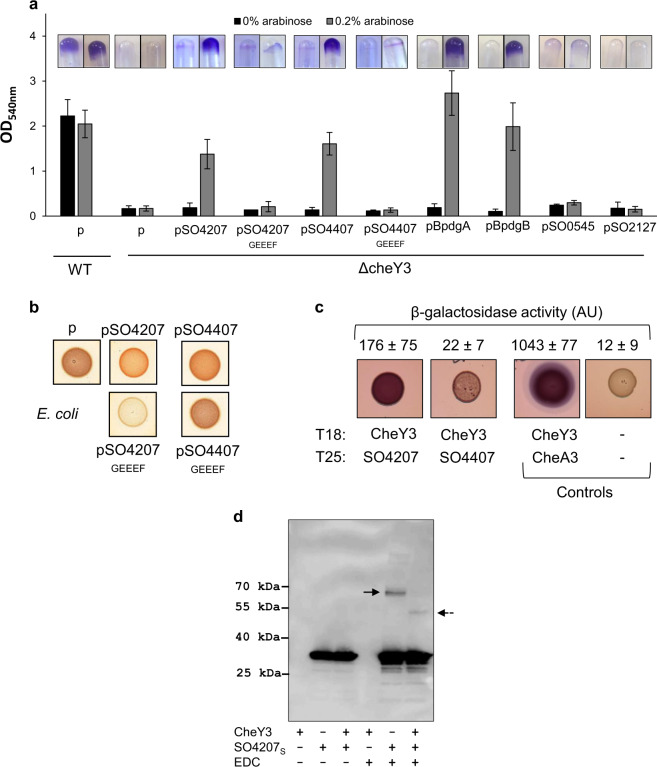
SSA-biofilm rescue of the *cheY3*-deleted mutant by overproduction of certain diguanylate cyclases. **a** Glass-tube biofilm assays of the wild-type and the ΔcheY3 strains containing either the empty vector (p), the pSO4207, pSO4207_GEEEF_, pSO4407, pSO4407_GEEEF_, pBpdgA, pBpdgB, pSO0545, or pSO2127 plasmids. Biofilms were grown with or without 0.2% arabinose for 24 h before CV-staining, imaging, and quantification. The means and standard deviations from at least two independent experiments conducted in duplicate are shown. **b** Congo red assays to test DGC activity. Cultures of *E. coli* cells containing either the empty vector, the pSO4207, pSO4207_GEEEF_, pSO4407, or pSO4407_GEEEF_ plasmids were spotted on LB plates containing Congo red and 0.2% arabinose. Plates were incubated for 2 days at 37 °C before imaging. All pictures were taken from the same plate and are representative of two independent experiments. **c** Two-hybrid assays to study CheY3-DGC interactions. Overnight cultures of *E. coli* cells producing proteins fused to the T25 domain (SO4207 and SO4407) and to the T18 domain (CheY3) of adenylate cyclase were spotted on MacConkey plates containing lactose. As controls, cells producing only the T18 and T25 domains (negative control) and cells producing the T18-CheY3 and the T25-CheA3 fusions (positive control) were spotted on the same plate. Plates were incubated for 2 days at 28 °C before imaging. All pictures were taken from the same plate and are representative of three independent experiments. The β-galactosidase activities were also measured on the same strains using a modified Miller assay and are indicated above the images (Miller arbitrary units (AU); mean values with standard deviations). **d** Cross-linking experiments between CheY3 and SO4207_S_. CheY3 (11.8 µM) and SO4207_S_ (3.1 µM) were incubated together or separately in the presence or absence of 5 mM EDC for 1 h at room temperature. The samples were then submitted to SDS-PAGE. After blotting, the membrane was revealed using a HisProbe-HRP conjugate (Thermo Fisher Scientific). The probable SO4207_S_ dimer is indicated by a solid arrow and the probable SO4207_S_-CheY3 complex is indicated by a dotted arrow.

We previously showed that CheY3 is able to interact with two diguanylate cyclases (DGCs), named PdgA and PdgB, in the context of pellicle formation. Given the crucial role played by CheY3 in SSA-biofilm biogenesis, we hypothesized that other DGCs could interact with CheY3 and be involved in SSA-biofilm formation. Therefore we applied the same strategy that was previously used in the pellicle context for SSA-biofilm recovery^40^. Briefly, we incubated the ΔcheY3 strain containing the genomic plasmid-borne library in LM medium containing lactate for 3 or 4 days. The cells that adhered to the tube walls were scratched away, resuspended in LM medium and then plated on LB-agar medium. Individual clones were then tested for their capabilities to form an SSA-biofilm. The plasmids contained in the biofilm-positive clones were extracted and the boundaries of their inserts were sequenced. Several clones were proven to contain at least one gene encoding a putative DGC (i.e., containing a GGDEF specific domain), namely SO4207 (three independent clones) and SO4407 (two independent clones). To ascertain that the SSA-biofilm rescue was due to these putative DGC-encoding genes, they were independently cloned under the control of the arabinose-inducible promoter and the resulting plasmids (pSO4207 and pSO4407) were introduced in the ΔcheY3 mutant strain. As shown in Fig. 4a, the presence of either pSO4207 or pSO4407 allowed SSA-biofilm formation in the ΔcheY3 background in the presence of the arabinose inducer. These results indicate that the overexpression of either SO4207 or SO4407 can partially restore SSA-biofilm formation in the *cheY3*-deleted strain and suggest that these two putative diguanylate cyclases play a role in SSA-biofilm biogenesis, probably by interacting with CheY3 and producing the secondary messenger c-di-GMP.

### SO4207 and SO4407 are active diguanylate cyclases, and one of them (SO4207) interacts with CheY3

The SO4207 and SO4407 proteins both contain a GGDEF domain which is characteristic of the DGCs. In addition, the N-terminal region of SO4207 harbors seven transmembrane segments and that of SO4407 contains a SnoaL_3 motif. Given that their active sites are both consensual (GGEEF), these two proteins should be able to synthesize c-di-GMP. To check this hypothesis, *Escherichia coli* cells containing the empty vector, the pSO4207 or the pSO4407 plasmid, were spotted on LB-agar plates containing Congo red and the arabinose inducer. As shown in Fig. 4b, cells containing either pSO4207 or pSO4407 were more red-colored than the cells with the empty vector, suggesting that SO4207 and SO4407 are capable of c-di-GMP synthesis when overexpressed in *E. coli*. To confirm these results, the consensual GGEEF motif of each DGC was changed to GEEEF and these inactive variants of the DGCs expressed in *E. coli* were also tested on Congo red plates. As shown in Fig. 4b, cells containing either the pSO4207_GEEEF_ or the pSO4407_GEEEF_ plasmid were less red-colored than cells with the corresponding wild-type plasmids, showing that SO4207 and SO4407 are capable of c-di-GMP synthesis and that their diguanylate activity requires the GGEEF motif as expected. In addition, these inactive variants were unable to restore SSA-biofilm formation in the *cheY3*-deleted strain (Fig. 4a). We thus conclude that the production of c-di-GMP by SO4207 and SO4407 is required for SSA-biofilm biogenesis.

We next wondered whether these two DGCs are able to interact with CheY3, as is the case for PdgA and PdgB. To test these putative interactions, we carried out bacterial two-hybrid assays using CheY3 fused to the T18 domain of adenylate cyclase and either the cytoplasmic domain of SO4207 or the SO4407 protein, fused to the T25 domain of adenylate cyclase. The cells containing T18-CheY3 and T25-SO4207 turned red on MacConkey-lactose plates and, accordingly, produced β-galactosidase to a high level (Fig. 4c). In contrast, when T18-CheY3 was combined with T25-SO4407, the cells were slightly red-colored. These results suggest that the diguanylate cyclase SO4207 interacts significantly with CheY3, while SO4407 interacts weakly with CheY3. To confirm the interaction between SO4207 and CheY3, we performed cross-linking experiments using a Strep-tagged CheY3 protein and a His-tagged soluble SO4207 protein devoid of the transmembrane segments. When the soluble domain of SO4207 (called thereafter SO4207_S_) was incubated alone in the presence of the cross-linker, a complex was observed and its molecular mass was in good agreement with the formation of a dimer (60 kDa) (Fig. 4d). When CheY3 (15 kDa) was incubated with SO4207_S_ (30 kDa), a CheY3-SO4207_S_ complex of about 45 kDa was observed (Fig. 4d).

We also tested the interaction between CheY3D56A or CheY3D56E and SO4207 by two-hybrid assays. The cells containing T18-CheY3D56E and T25-SO4207 were redder than that containing T18-CheY3D56A and T25-SO4207 (Supplementary Fig. 1a). This result is in agreement with the fact that CheY3 must be phosphorylated for SSA-biofilm biogenesis. By using a similar approach, we showed that neither SO4207 nor SO4407 interacts with the c-di-GMP effector MxdA (Supplementary Fig. 1b). These 2 DGCs could therefore stimulate another still unknown effector protein.

**Fig. 5:**
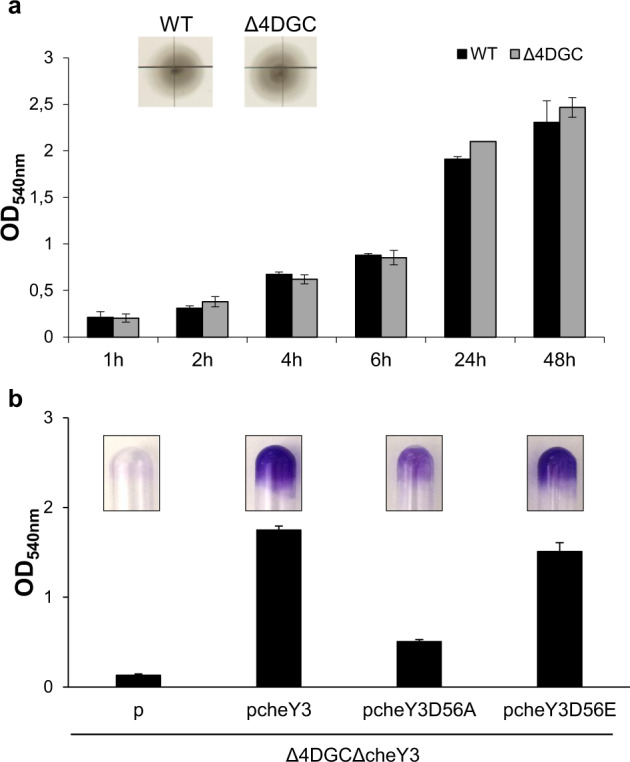
The Δ4DGC mutant is still able to form an SSA-biofilm. **a** Kinetics of biofilm formation of the wild-type strain (WT) and the Δ4DGC mutant strain (deleted for four DGC-encoding genes: *pdgA*, *pdgB*, SO4207, and SO4407). Biofilms were grown for 1, 2, 4, 6, 24, and 48 h before CV-staining and quantification. The insert corresponds to swim assays of the wild-type and the Δ4DGC strains. Cells were grown overnight on LB plates and stabbed on tryptone-plates containing 0.25% agar. Pictures were taken from the same plate after 2 days of incubation at 28 °C. **b** Glass-tube biofilm assays of the Δ4DGCΔcheY3 mutant strain containing either the empty vector (p) or the pcheY3 WT or mutant plasmids. Biofilms were grown 24 h with 0.2% arabinose before CV-staining, imaging, and quantification. The means and standard deviations from two independent experiments conducted in duplicate are shown.

Since the overexpression of either the SO4207 or the SO4407 gene at least partially overcomes the failure of the *cheY3*-deleted strain to form an SSA-biofilm, we tested whether PdgA and PdgB also contribute to SSA-biofilm formation. Interestingly, overexpression of either PdgA or PdgB allowed the ΔcheY3 to form an SSA-biofilm (Fig. 4a). We then wanted to determine whether the deletion of the four DGCs could affect SSA-biofilm biogenesis. The strain named Δ4DGC (deleted for *pdgA*, *pdgB*, SO4207, and SO4407) was grown in the SSA-biofilm condition and its capability to adhere to the tube walls was estimated at different time points by CV-staining. We also tested the chemotactic behavior of this mutant. Unfortunately, the behavior of the Δ4DGC was similar to that of the wild-type strain both for SSA-biofilm formation and chemotaxis (Fig. 5a). This result clearly indicates that other DGCs among the 50 putative ones encoded by the *S. oneidensis* genome are probably implicated in SSA-biofilm formation. In agreement with this hypothesis, overexpression of either wild-type *cheY3* or *cheY3D56E* but not *cheY3D56A* could restore SSA-biofilm formation in the Δ4DGCΔcheY3 mutant, indicating that phosphorylated CheY3 is mandatory for SSA-biofilm even in the absence of the four DGCs (Fig. 5b). In an attempt to identify other DGCs involved in SSA-biofilm biogenesis, we reasoned that phosphorylation could be important for the DGCs as for CheY3. Among the 50 putative DGCs of *S. oneidensis*, only two contain a receiver domain with a phosphorylatable aspartate residue (SO0545 and SO2127). These two DGCs were therefore overexpressed in the ΔcheY3 mutant, but they failed to restore SSA-biofilm formation in this mutant context, suggesting that they are not involved in this process (Fig. 4a).

### Two orthologous CheY proteins can replace CheY3 for SSA-biofilm formation but not for chemotaxis

**Fig. 6:**
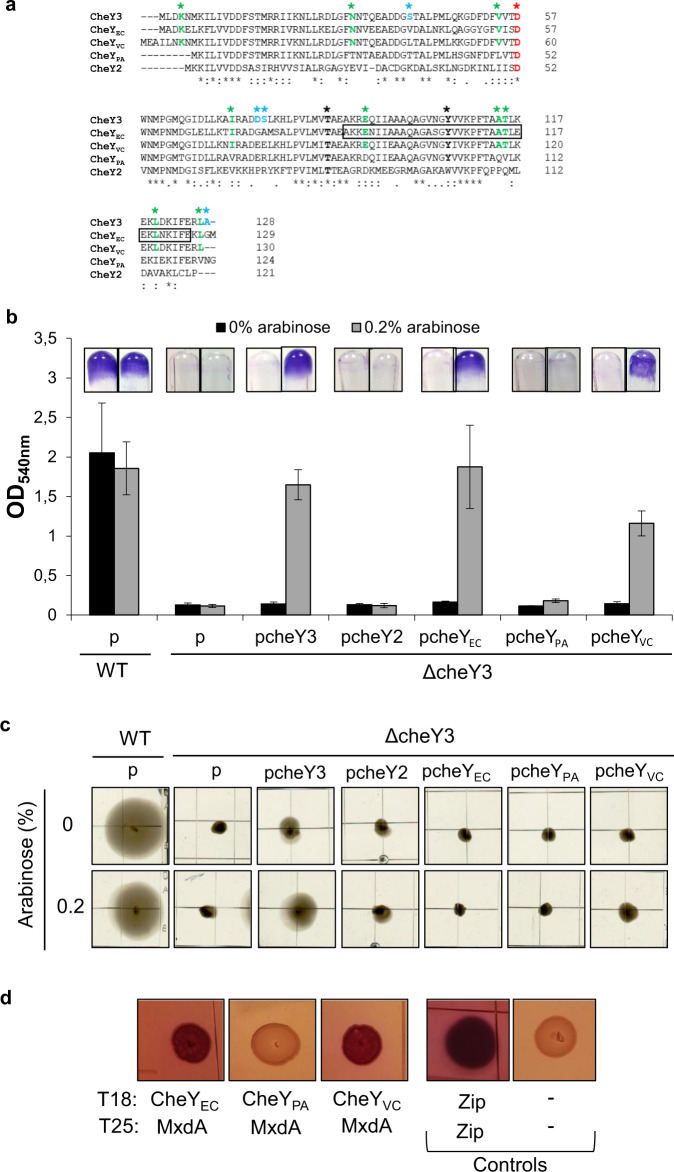
Overexpression of CheY-encoding genes from *E. coli* and *V. cholerae* restores SSA-biofilm formation into the *cheY3*-deleted strain. **a** Amino acid sequence alignments of CheY proteins from *Shewanella oneidensis* (CheY3 and CheY2), *Escherichia coli* (CheY_EC_), *Pseudomonas aeruginosa* (CheY_PA_), and *Vibrio cholerae* (CheY_VC_). The conserved phosphorylatable aspartate residue of each protein is in red (also indicated by a red asterisk). Residues found only in CheY3, CheY_EC_, and CheY_VC_ are in green (also indicated by green asterisks). Residues found only in CheY3 are in blue (also indicated by blue asterisks). The Thr87 and Tyr106 of CheY_EC_, which are displaced upon CheY phosphorylation, as well as the corresponding conserved sites in the other CheY, are in bold (also indicated by black asterisks). The region of CheY_EC_, which is involved in the interaction with FliM, is indicated by a black box^65^. **b** Glass-tube biofilm assays of the wild-type and the ΔcheY3 strains containing either the empty vector (p) or plasmids bearing CheY-encoding genes from *S. oneidensis* (pcheY3 and pcheY2)*, E. coli* (pcheY_EC_)*, P. aeruginosa* (pcheY_PA_), or *V. cholerae* (pcheY_VC_). Cells were grown in the presence or absence of arabinose for 24 h before CV-staining, imaging, and quantification. The means and standard deviations from two independent experiments conducted in duplicate are shown. **c** Swim assays of the wild-type and the ΔcheY3 strains containing either the empty vector (p), the pcheY3, pcheY2, pcheY_EC_, pcheY_PA_, or pcheY_VC_ plasmids. Cells were grown overnight on LB plates and stabbed on tryptone-plates containing 0.25% agar in the presence or absence of arabinose. All pictures were taken from the same plate after 2 days of incubation at 28 °C and are representative of three independent experiments. **d** Two-hybrid assays to study interactions of CheY3 homologs with MxdA. Overnight cultures of *E. coli* cells producing proteins fused to the T25 domain (MxdA) and to the T18 domain (CheY_EC_, CheY_PA_, or CheY_VC_) of adenylate cyclase were spotted on MacConkey plates containing lactose. As controls, cells producing only the T18 and T25 domains (negative control) and cells producing the T18-zip and the T25-zip fusions (positive control) were spotted on the same plate. Plates were incubated for 2 days at 28 °C before imaging. All pictures were taken from the same plate and are representative of two independent experiments.

As CheY3 plays a key role in SSA-biofilm development, we wondered whether orthologous chemotaxis *cheY* genes could complement the *cheY3*-deleted strain for SSA-biofilm formation and for chemotaxis. To test this, we chose the chemotaxis CheY proteins from *E. coli*, *P. aeruginosa*, and *Vibrio cholerae* which present various degrees of similarity with CheY3 (Fig. 6a). As a control, we also selected the CheY2 protein (SO2318), which belongs to a non-chemotactic Che-like system and obviously presents a lower degree of similarity with the chemotaxis CheY proteins (Fig. 6a). The corresponding *cheY* genes were cloned under the control of the arabinose-inducible promoter and the resulting plasmids (pcheY_EC_, pcheY_PA_, pcheY_VC_, and pcheY2) were introduced into the ΔcheY3 strain. We then tested the different strains for their capabilities to form an SSA-biofilm and for their chemotactic behavior. As shown in Fig. 6b, the presence of either pcheY_EC_ or pcheY_VC_ allowed SSA-biofilm formation in the ΔcheY3 background in the presence of the arabinose inducer. It should be noted that the presence of CheY_EC_ allowed biofilm formation to a level comparable to that of the wild-type strain, while the complementation by CheY_VC_ was slightly less efficient. In contrast, neither pcheY_PA_ nor pcheY2 was able to complement the ΔcheY3 strain for biofilm formation (Fig. 6b). These results indicate that at least two orthologous chemotaxis CheY regulators can replace CheY3 for SSA-biofilm development.

The same strains were then spotted on soft-agar plates. As expected, the ΔcheY3 strain was non-chemotactic and the presence of the wild-type *cheY3* gene, but not that of the *cheY2* gene, was able to restore its chemotactic behavior (Fig. 6c). Surprisingly, none of the orthologous chemotaxis *cheY* genes were able to overcome the chemotactic default of the ΔcheY3 strain. Together, these results show that CheY_EC_ and CheY_VC_ can replace CheY3 for SSA-biofilm formation but not for chemotaxis, meaning that the two functions of CheY3 can be dissociated and probably involve distinct regions of the protein. Surprisingly, CheY_PA_ does not differ significantly from CheY3, CheY_EC_, and CheY_VC_. Subtle modifications, in particular in the region affected by phosphorylation, could be involved in *cheY3* complementation (Fig. 6a).

### Like CheY3, CheY_EC_ and CheY_VC_ interact with the c-di-GMP effector MxdA

It was previously shown that the Mxd machinery, which is probably involved in the synthesis and secretion of an exopolysaccharide, was required for SSA-biofilm development^34,37^. As it was recently demonstrated that CheY3 interacts with the c-di-GMP effector MxdA^40^, which probably activates the Mxd machinery, we wanted to determine whether the orthologous CheY proteins could bind MxdA. We therefore performed bacterial two-hybrid assays using MxdA fused to the T25 domain of adenylate cyclase and the CheY proteins fused to the T18 domain of adenylate cyclase. As shown in Fig. 6d, the cells containing T25-MxdA and either T18-CheY_EC_ or T18-CheY_VC_ turned red on MacConkey-lactose plates. On the contrary, when T25-MxdA was combined with T18-CheY_PA_, the cells did not turn red (Fig. 6d). These results indicate that CheY_EC_ and CheY_VC_ interact with MxdA, while CheY_PA_ does not. Strikingly, this correlates well with the fact that only CheY_EC_ and CheY_VC_ were able to replace CheY3 for SSA-biofilm formation. Altogether, these results strongly suggest that the interaction between CheY and MxdA is crucial for SSA-biofilm biogenesis.

## Discussion

Our results reveal intriguing features. First, CheY is the main regulator of chemotaxis and is well known to interact upon phosphorylation with the rotor of the flagellum to change or stop its rotation. CheY3 of *S. oneidensis* is essential not only during chemotaxis but also for the biogenesis of the SSA-biofilm (this work) and of the pellicle^32^. Second, although CheY3 must be phosphorylated to allow the formation of SSA-biofilm, CheA3, its dedicated kinase for chemotaxis, is not involved in CheY3 phosphorylation during biofilm development. However, CheA3 seems to limit the thickness of the biofilm by an unknown mechanism. Still unknown kinases, specifically involved in biofilm induction, probably phosphorylate CheY3 when necessary. In contrast, CheA3 plays a major role during pellicle maturation^32^. Third, the CheY chemotaxis regulator usually interacts solely with its kinase (CheA), phosphatase (CheZ or other), and the rotor of the flagellum. We have shown that CheY3 could also bind several diguanylate cyclases (DGCs) and c-di-GMP effectors, including MxdA and probably others. Finally, to our surprise, certain CheY regulators of other bacteria can replace CheY3 for biofilm formation but not for chemotaxis. These data confirm that the two functions of CheY3 are distinct.

Our proposal is that CheY3-P could connect DGCs to the c-di-GMP effectors in line with the idea that c-di-GMP is delivered directly to the target effector. CheY3-P might also trigger the enzymatic activity of certain DGCs. Indeed, the phosphorylation of the DGC itself or of an additional binding partner was previously shown to be involved in the control of DGC activity^41–44^. The phosphorylation of CheY regulators partially modifies the structure of their C-terminal subdomains^45–47^. We thus propose that the phosphorylation of CheY3 unmasks specific regions of interaction with both DGCs and c-di-GMP effectors. Biochemical and structural analyses are now required to define these regions and to understand, at the molecular level, the mechanism of action of CheY3-P during the biogenesis of SSA-biofilm. Indeed, as shown in the paper, CheY3-P plays a key role throughout biofilm formation, meaning that its role is not restricted to a single phase of biofilm development. In the literature, the involvement of the flagellum during the attachment step is well documented, whereas a possible role of the chemotaxis regulator CheY has been poorly studied^48–50^. However, the involvement of Che or Che-like systems in building biofilms has been reported several times^51–57^. The fact that orthologous CheY proteins can suppress the defect of biofilm synthesis in a *cheY3* mutant suggests a probable dual role of CheY in other bacteria. It is not surprising that, at least for bacteria from or close to the *Shewanella* genus, the CheY chemotaxis regulator could also act as a post-translational regulator of biofilm synthesis.

In *S. oneidensis*, transcriptional regulation of biofilm formation is probably under the control of FlrA, a homolog of FleQ, the regulator of many genes involved in biofilm control in *P. aeruginosa*. FleQ acts first as a repressor, but repression is released in the presence of c-di-GMP^12^. The c-di-GMP-binding motifs of FleQ are well conserved within FlrA in agreement with a similar role of FleQ and FlrA in their respective strains, as demonstrated for FlrA in *Shewanella putrefaciens* CN32^58^. For instance, genes encoding DGCs implicated in biofilm development could be regulated by FlrA.

Of interest, the genome of *S. oneidensis* encodes 50 DGCs and we have shown that deletion of the four genes encoding DGCs known to be involved in biofilm synthesis did not abrogate the formation of SSA-biofilm. This result indicates that several additional DGCs need to be discovered. A library devoid of the four DGC-encoding genes will help to identify new biofilm DGCs. Alternatively, CheY3D56E, the phospho-mimetic CheY3 protein, could be used as a bait to find new DGCs and c-di-GMP effectors after a two-hybrid screen or co-purification. Induction of DGC genes during biofilm biogenesis might also reveal DGCs involved in biofilm development.

In conclusion, this study revealed the unsuspected role of a chemotaxis regulator in biofilm biogenesis. The regulation of biofilm development is a complex process including many signals activating DGCs. As CheY3 must be phosphorylated, probably by histidine kinases, CheY3 and its connected kinases add another layer to the control of biofilm formation. It thus opens new attractive avenues in the field of the post-translational regulation of biofilm generation.

## Methods

### Strains and growth conditions

All strains used in this study are listed in Table 1 and were routinely grown at 28 °C (*S. oneidensis* strains) or 37 °C (*E. coli* strains) in Lysogeny Broth (LB) medium. When needed, antibiotics were used at 10 μg.ml^−1^ (rifampicin), 25 μg.ml^−1^ (chloramphenicol and kanamycin), 50 μg.ml^−1^ (ampicillin), or 100 μg.ml^−1^ (streptomycin). Arabinose was used at 0.2%.

**Table 1:** Strains used in this study.

Strains	Genotypes	References
*S. oneidensis*		
MR1-R	Rifampicin-resistant derivative of MR1 (referred as the wild-type)	^66^
ΔcheA3	MR1-R deleted of *cheA3* (SO3207)	^32^
ΔcheY3	MR1-R deleted of *cheY3* (SO3209)	^32^
ΔcheA3ΔcheY3	MR1-R deleted of *cheA3* (SO3207) and *cheY3* (SO3209)	This work
Δ4DGC	MR1-R deleted of *pdgA* (SO4552), *pdgB* (SO0796), SO4207, and SO4407	This work
Δ4DGCΔcheY3	MR1-R deleted of *pdgA* (SO4552), *pdgB* (SO0796), SO4207, SO4407, and *cheY3* (SO3209)	This work
*E. coli*		
C600	F*- tonA21 thi-1 thr-1 leuB6 lacY1 glnV44 rfbC1 fhuA1 λ-*	^67^
CC118 λpir	*Δ(ara-leu) araDE ΔlacX74 galE galK phoA20 thi-1 rpsE rpoB argE* (Am) *recA1* λpir	^59^
BTH101	F- *cya-99 araD139 galE15 galK16 rpsL1* (Str^R^) *hsdR2 mcrA1 mcrB1*	^61^
BL21 (DE3)	F- *ompT hsdSB (rB-mB-) dcm gal* (DE3)	Novagen

### Construction of deletion mutants

To construct the ΔcheA3ΔcheY3 mutant strain, the *cheY3* (SO3209) gene was deleted from the ΔcheA3 genome^32^. The Δ4DGC quadruple mutant strain was constructed by sequential deletion of the SO4207 and SO4407 genes, starting with the ΔpdgAΔpdgB double mutant strain^40^. To construct the Δ4DGCΔcheY3 mutant strain, the *cheY3* gene was deleted from the Δ4DGC genome. These deletion mutants were constructed as follows. Upstream and downstream 500 bp-regions flanking the gene to be deleted (SO3209, SO4207, and SO4407) were fused and cloned into the suicide vector pKNG101. The ligation product was introduced into *E. coli* CC118 λpir^59^. The resulting plasmid was introduced into the appropriate *S. oneidensis* strain by conjugation using the *E. coli* helper strain 1047/pRK2013^60^. The plasmid was integrated in the chromosome by a first recombination event and removed by a second recombination event in the presence of 6% sucrose. Deletions were confirmed by PCR.

### Construction of plasmids

All plasmids used in this study are listed in Table 2. To construct pSO4207, pSO4407, pSO0545, pSO2127, and pcheY2 plasmids, the coding sequences of SO4207, SO4407, SO0545, SO2127, and SO2318, respectively, were PCR-amplified using chromosomal *S. oneidensis* DNA as a template and primers containing appropriate restriction sites and an optimized Shine Dalgarno. After digestion, the PCR products were inserted into the pBAD33 vector. To construct the pSO4207_GEEEF_ and pSO4407_GEEEF_ plasmids, the pSO4207 and pSO4407 plasmids were amplified by PCR using appropriate primers, following the protocol of the Q5 Site-directed Mutagenesis kit (NEB). The pcheY3D56E plasmid was constructed by PCR using mutagenic primers divergently orientated and overlapping at their 5′ ends, and pcheY3 DNA as template. To construct pcheY_EC_, pcheY_PA_, and pcheY_VC_ plasmids, the coding sequences of GBKW3110_3069 from *E. coli* W3110, PA1456 from *P. aeruginosa* PAO1, and VC0395_A1653 from *V. cholerae* O395, respectively, were PCR-amplified using primers containing appropriate restriction sites and an optimized Shine Dalgarno. After digestion, the PCR products were inserted into the pBAD33 vector.

**Table 2:** Plasmids used in this study.

Plasmids	Descriptions	References
pX5-GFP	Sequence coding for GFP cloned into pX5 vector	^68^
pBAD33	Vector containing pBAD promoter with the p15A origin of replication	^69^
pcheY3	*cheY3* (SO3209) sequence cloned into pBAD33	^32^
pcheY3D56A	*cheY3* (SO3209) sequence with the point mutation D56A cloned into pBAD33	^32^
pcheY3D56E	*cheY3* (SO3209) sequence with the point mutation D56E cloned into pBAD33	This work
pcheY2	*cheY2* (SO2318) sequence cloned into pBAD33	This work
pcheY_EC_	GBKW3110_3069 sequence cloned into pBAD33	This work
pcheY_PA_	PA1456 sequence cloned into pBAD33	This work
pcheY_VC_	VC0395_A1653 sequence cloned into pBAD33	This work
pSO4207	SO4207 sequence cloned into pBAD33	This work
pSO4407	SO4407 sequence cloned into pBAD33	This work
pSO4207_GEEEF_	SO4207 sequence with the point mutation G535E cloned into pBAD33	This work
pSO4407_GEEEF_	SO4407 sequence with the point mutation G254E cloned into pBAD33	This work
pBpdgA	*pdgA* (SO4552) sequence cloned into pBAD33	^40^
pBpdgB	*pdgB* (SO0796) sequence cloned into pBAD33	^40^
pSO0545	SO0545 sequence cloned into pBAD33	This work
pSO2127	SO2127 sequence cloned into pBAD33	This work
pEB355	pUT18C derivative, coding for the T18 domain of the adenylate cyclase of *Bordetella pertussis*	^61^
pUT18-cheY3	*cheY3* (SO3209) sequence cloned in-frame at the 3′ end of the sequence coding for the T18 domain into pEB355	^40^
pUT18-cheY3D56A	*cheY3D56A* sequence cloned in-frame at the 3′ end of the sequence coding for the T18 domain into pEB355	This work
pUT18-cheY3D56E	*cheY3D56E* sequence cloned in-frame at the 3′ end of the sequence coding for the T18 domain into pEB355	This work
pUT18-cheY_EC_	GBKW3110_3069 sequence cloned in-frame at the 3′ end of the sequence coding for the T18 domain into pEB355	This work
pUT18-cheY_PA_	PA1456 sequence cloned in-frame at the 3′ end of the sequence coding for the T18 domain into pEB355	This work
pUT18-cheY_VC_	VC0395_A1653 sequence cloned in-frame at the 3′ end of the sequence coding for the T18 domain into pEB355	This work
pUT18-mxdA	*mxdA* (SO4180) sequence cloned in-frame at the 3′ end of the sequence coding for the T18 domain into pEB355	This work
pT18-zip	Sequence coding for a leucine zipper region cloned in-frame with the T18 domain (positive control)	^61^
pEB354	pKT25 derivative, coding for the T25 domain of the adenylate cyclase of *Bordetella pertussis*	^61^
pKT25-cheA3	*cheA3* (SO3207) sequence cloned in-frame at the 3′ end of the sequence coding for the T25 domain into pEB354	^40^
pKT25-mxdA	*mxdA* (SO4180) sequence cloned in-frame at the 3′ end of the sequence coding for the T25 domain into pEB354	^40^
pKT25-SO4207	Sequence coding for the cytoplasmic domain of SO4207 (D388 to N625) cloned in-frame at the 3′ end of the sequence coding for the T25 domain into pEB354	This work
pKT25-SO4407	SO4407 sequence cloned in-frame at the 3′ end of the sequence coding for the T25 domain into pEB354	This work
pT25-zip	Sequence coding for a leucine zipper region cloned in-frame with the T25 domain (positive control)	^61^
pETcheY3	Sequence coding for CheY3 (SO3209) cloned into pET52b	^40^
pET-SO4207_S_	Sequence coding for the cytoplasmic domain (D388 to N625) of SO4207 cloned into pET21b	This work
pKNG101	R6K-derived suicide plasmid containing Str^R^ and *sacB*	^59^
pRK2013	RK2-Tra1 RK2-Mob1 Km^R^ *ori* ColE1	^60^

To construct the plasmid pET-SO4207_S_ allowing the overproduction of the cytoplasmic domain of SO4207 (D388 to N625) fused to a 6xHis-tag, the sequence coding for the cytoplasmic domain of SO4207 was amplified from *S. oneidensis* genomic DNA by PCR and cloned into pET21b with sequence coding for 6xHis-tag downstream on the vector (Novagen).

For two-hybrid experiments, the sequence coding for the cytoplasmic domain of SO4207 (D388 to N625) and the SO4407 coding sequence from *S. oneidensis* were cloned in-frame at the 3′ end of the sequence coding for the T25 domain of adenylate cyclase into pEB354^61^, leading to pKT25-SO4207 and pKT25-SO4407, respectively. The GBKW3110_3069 (from *E. coli* W3110), PA1456 (from *P. aeruginosa* PAO1), VC0395_A1653 (from *V. cholerae* O395), and MxdA (from *S. oneidensis*) coding sequences were cloned in-frame at the 3′ end of the sequence coding for the T18 domain of adenylate cyclase into pEB355^61^ leading to pUT18-cheY_EC_, pUT18-cheY_PA_, pUT18-cheY_VC_, and pUT18-mxdA, respectively. To construct the pUT18-cheY3D56A and pUT18-cheY3D56E plasmids, the pUT18-cheY3 plasmid was amplified by PCR using appropriate primers, following the protocol of the QuickChange Site-directed Mutagenesis kit (Agilent).

All constructs were checked by DNA sequencing using appropriate primers.

### Biofilm assays in glass tubes

Biofilm formation in borosilicate glass tubes was performed as follows. Cells were grown overnight on LB-agar plates at 28 °C. Cells were then gently resuspended in LM medium (0.2 g.l^−1^ yeast extract, 0.1 g.l^−1^ peptone, 10 mM HEPES (pH 7.4), and 10 mM NaHCO_3_) containing 20 mM lactate and diluted in the same medium to reach an OD_600nm_ of 0.05. Two milliliters of the cell dilutions were transferred into borosilicate glass tubes and incubated with shaking at 28 °C for the indicated time. The liquid medium was carefully removed from the tubes and the bacterial cells bound to the walls were stained with 3.5 ml of 0.2% crystal violet (CV) for 10 min. Tubes were rinsed several times with water to remove unbounded crystal violet and imaged. For spectrophotometric quantification, the dye was solubilized in 3 ml of 30% acetic acid and the OD was measured at 540 nm. Strains containing plasmids were grown in the presence of chloramphenicol. Arabinose (0.2%) was added when needed during the biofilm assay. When CV-measurements were performed during the early steps of SSA-biofilm formation (i.e., from 1 to 8 h of incubation), arabinose was also added during the overnight culture.

### Kinetics assays of SSA-biofilm formation

To study the kinetics of SSA-biofilm formation of the ΔcheY3/pcheY3D56E strain, cells were grown overnight on LB plates containing chloramphenicol and arabinose. Cells were used to carry out a biofilm assay in glass tubes (see above) in the presence of arabinose (pre-incubation). After 2, 4, and 8 h of pre-incubation, three tubes were recovered: one tube was CV-stained and the other two tubes were emptied and gently washed to remove unbound cells. The latter 2 tubes were filled with ΔcheY3/pcheY3D56E cells grown overnight on plates and diluted in LM medium containing lactate (final OD_600nm_ = 0.05). For one tube, both overnight growth and resuspension in LM medium were performed in the presence of arabinose, while overnight growth and resuspension in LM medium were performed without arabinose for the other tube. These 2 tubes were then incubated under SSA-biofilm formation. Twenty-four hours after the start of the pre-incubation, all tubes were CV-stained and SSA-biofilm was quantified as described above.

### Flow cell biofilm assays

Time-lapse biofilm formation was further performed in flow chambers with individual channel dimensions of 1 × 4 × 40 mm. To study the biofilm formation of *S. oneidensis* strains in three-channel flow cells, cells containing pX5-GFP plasmid were grown overnight on LB-agar plates containing chloramphenicol at 28 °C. Cells were resuspended in LM medium containing 20 mM lactate and diluted in the same medium to reach an OD_600nm_ of 0.01. Three hundred microliters of cell dilutions were injected into each channel. The chamber was then inverted and incubated for 3 h at 28 °C to enable initial cell adhesion to the glass slide, after which flow was initiated with a Watson Marlow 205S peristaltic pump. Each channel was supplied with 3 ml.h^−1^ of LM medium containing 20 mM lactate and biofilms were grown at 30 °C. The mean flow velocity in the flow cells was 0.2 mm.s^−1^. After 24 h of incubation, the biofilm was observed by confocal laser scanning microscopy with an Olympus FV-1000 microscope equipped with detectors and filter sets for monitoring of GFP. All confocal images were analyzed using the imageJ software^62^.

### Testing of the ΔcheY3 strain containing the genomic library under SSA-biofilm conditions

Two aliquots (containing about 1.5 × 10^9^ cells each) of the ΔcheY3 strain containing the plasmid library of *S. oneidensis* chromosomal fragments^40^ were centrifuged, resuspended with 1 ml LB and incubated at 28 °C for 2 h under shaking conditions. The two cultures were then centrifuged and resuspended with 2 mL LM medium containing 20 mM lactate and 0.2% arabinose. The two resulting cultures were transferred into borosilicate glass tubes and incubated at 28 °C under static conditions. After 3 days of incubation for the first aliquot and 4 days for the second, the medium containing planktonic cells was carefully removed and the tubes were gently washed with LM medium to remove unbound cells. Most of the cells that adhered to the tube walls were detached with a sterile inoculating loop, resuspended in 1 ml LM medium, and serial-diluted. Cells that adhered to the glass walls located at the air–liquid interface were not recovered because they are theoretically part of the pellicle and not of the SSA-biofilm. Cells that remained attached to the tube walls were CV-stained to check that SSA-biofilm formation had occurred before cell dilutions were spread on plates containing chloramphenicol. Clones were PCR-tested using *cheY3*, *pdgA*, and *pdgB* specific primers. Clones that did not contain *cheY3*, p*dgA*, and *pdgB* were used to carry out an SSA-biofilm assay in 24-well plates under static conditions in the presence of 0.2% arabinose. Plasmids of the clones that had formed an SSA-biofilm were extracted and sequenced using primers that hybridized on both sides of the vector cloning site.

### Congo red assays

Diguanylate cyclase activities were measured following the protocol developed by De et al.^63^. *E. coli* C600 cells containing either the empty vector (pBAD33) or a pBAD33-derived plasmid (pSO4207, pSO4407, pSO4207_GEEEF_, pSO4407_GEEEF_) were grown under shaking conditions in LB medium containing chloramphenicol at 37 °C until an OD_600nm_ of about 0.6 was reached. Subsequently, 10 μl bacterial cultures were spotted onto LB plates containing chloramphenicol and 50 µg.ml^−1^ Congo red, along with, when indicated, 0.2% of arabinose. Pictures were taken after 2 days of incubation at 37 °C.

### Swim plate assays

Cells were grown overnight on LB plates and stabbed with a sterile toothpick on tryptone swim plates (10 g.l^−1^ tryptone, 5 g.l^−1^ NaCl, and 0.25% soft agar) containing 0.2% arabinose when indicated. Swim plates were incubated for 2 days at 28 °C before imaging. Strains containing pBAD33-derived plasmids were grown overnight in the presence of chloramphenicol.

### Bacterial two-hybrid assays

Bacterial two-hybrid assays were performed as follows. The reporter strain *E. coli* BTH101 (*cya*) was co-transformed with two-hybrid plasmids. For negative controls, pEB354 and pEB355 vectors were used. For positive controls, either the pT18-zip and pT25-zip, pUT18-cheY3 and pKT25-cheA3, or pUT18-mxdA and pKT25-mxdA plasmids were used. Clones were selected on LB-agar plates containing 50 µg.ml^−1^ ampicillin and 25 µg.ml^−1^ kanamycin and incubated at 28 °C for 4 days. Ten clones of each co-transformation were inoculated in LB medium containing ampicillin, kanamycin, and 0.5 mM IPTG and grown overnight at 28 °C under shaking conditions. Interactions were detected using colorimetric assays and/or β-galactosidase assays. For colorimetric assays, two microliters of each overnight culture were spotted onto MacConkey plates with 1% lactose (Difco^TM^ MacConkey agar), ampicillin, and kanamycin. Plates were incubated for 2 days at 28 °C before imaging. For β-galactosidase assays, cells were lysed for 15 min using lysozyme and PopCulture reagent (Agilent) before addition of Z buffer (100 mM phosphate buffer pH 7, 10 mM KCl, 1 mM MgSO_4_, 50 mM β-mercaptoethanol). Then, 2.2 mM ortho-nitrophenyl-β-galactoside (ONPG) was added to the mix and kinetic quantifications of OD_420nm_ were performed using a Tecan Spark microplate reader^64^. Slope of the obtained curves was calculated and β-galactosidase activity (arbitrary units) was determined by the following equation: (1000 × slope)/(OD_600nm_ × volume of reaction (µL)).

### Expression and purification of recombinant proteins

The Strep-tagged CheY3 protein was produced using BL21 (DE3) cells containing pETcheY3 and purified on Strep-Tactin resin (IBA) according to the manufacturer’s protocol^40^. To produce His-tagged SO4207_S_ protein, BL21 (DE3) strain containing pET-SO4207_S_ was grown aerobically at 37 °C until OD_600_ reaches 0.8 units. Protein overproduction was then induced with 0.1 mM isopropyl β-D-thiogalactopyranoside (IPTG) for 20 h at 28 °C. Cells were then harvested by centrifugation, resuspended in phosphate buffer (20 mM sodium phosphate (pH 7.4), 0.5 M NaCl) and disrupted by French press. Lysates were centrifuged 20 min at 15,500 × *g* and the supernatants were centrifuged 1 h at 185,600 × *g*. The resulting supernatants were then loaded onto a HisTrapFF column (GE Healthcare) and protein was purified according to the manufacturer’s protocol. Proteins were stored at −80 °C in elution buffer containing 10% glycerol.

### Chemical cross-linking experiments

Cross-linking experiments were performed as follows. Strep-tagged CheY3 (11.8 µM) and His-tagged SO4207_S_ (3.1 µM) were incubated for 1 h at room temperature in 10 mM cacodylate buffer containing 5 mM 1-Ethyl-3-(3-dimethylaminopropyl) carbodiimide hydrochloride (EDC). The reactions were stopped by adding 50 mM Tris pH8. The interactions were analyzed by SDS-PAGE followed by Western blotting using HisProbe^TM^-HRP conjugate (Thermo Fisher Scientific). All samples were processed on the same blot. The original full-length blot is shown in the Supplementary Figures file.

### Sequence alignments

Amino acid sequences of CheY3, CheY2, CheY_EC_, CheY_PA_, and CheY_VC_ were aligned using the Clustal Omega program (European Molecular Biology Laboratory) with default parameters.

### Reporting summary

Further information on research design is available in the Nature Research Reporting Summary linked to this article.

## Supplementary information

Supplementary Information

Reporting Summary Checklist

## Data Availability

The authors declare that all relevant data supporting the findings of the study are available in this article and the Supplementary Figures file, or from the corresponding author upon reasonable request.
