# Supplementary material for: The phosphorylated regulator of chemotaxis is crucial throughout biofilm biogenesis in Shewanella oneidensis
Source: NPJ Biofilms Microbiomes. 2020 Nov 13;6:54. doi: 10.1038/s41522-020-00165-5 (PMC7666153; doi:10.1038/s41522-020-00165-5)
Supplement: Supplementary file 1 — Supplementary Information [file 41522_2020_165_MOESM1_ESM.pdf]

a

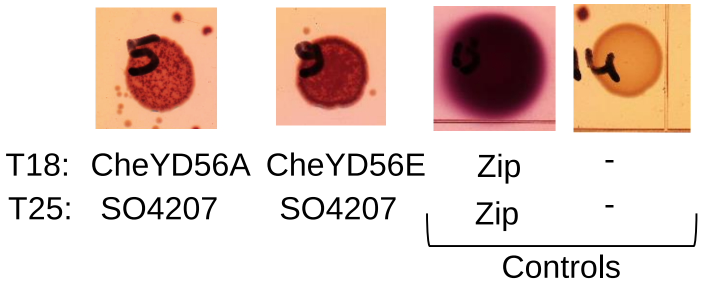

b

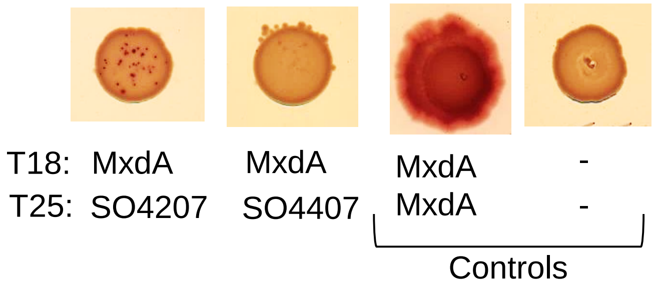

Supplementary Fig. 1. Two-hybrid assays. a Overnight cultures of *E. coli* cells producing proteins fused to the T25 domain (SO4207) and to the T18 domain (CheY3D56A and CheY3D56E) of adenylate cyclase were spotted on MacConkey plates containing lactose. As controls, cells producing only the T18 and T25 domains (negative control) and cells producing the T18-Zip and the T25-Zip fusions (positive control) were spotted on the same plate. Plates were incubated for 2 days at 28°C before imaging. **b** Overnight cultures of *E. coli* cells producing proteins fused to the T25 domain (SO4207 and SO4407) and to the T18 domain (MxdA) of adenylate cyclase were spotted on MacConkey plates containing lactose. As controls, cells producing only the T18 and T25 domains (negative control) and cells producing the T18-MxdA and the T25-MxdA fusions (positive control) were spotted on the same plate. Plates were incubated for 2 days at 28°C before imaging.

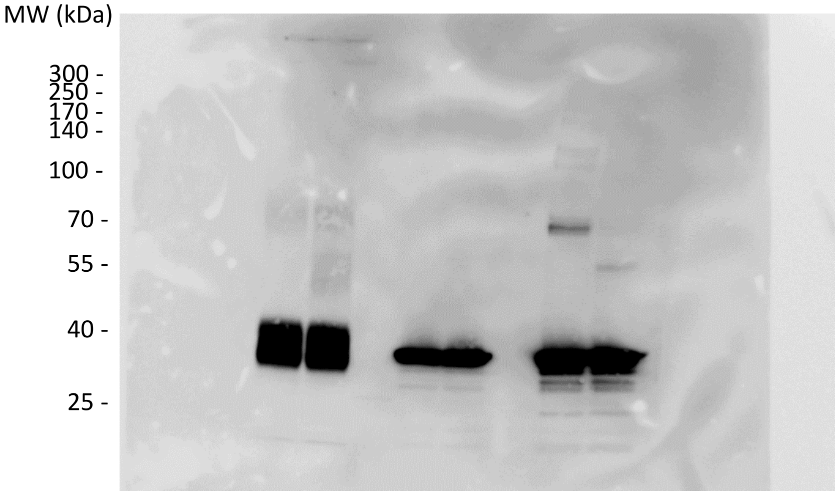

Full-length original blot shown in figure 4d
